# Dynamic Perturbations of CD4 and CD8 T Cell Receptor Repertoires in Chronic Hepatitis B Patients upon Oral Antiviral Therapy

**DOI:** 10.3389/fimmu.2017.01142

**Published:** 2017-09-14

**Authors:** Ying Xu, Yu Liu, Miaoxian Zhao, Yunqing Chen, Cantao Xie, Mingxing Gong, Haohui Deng, Xueying Li, Jian Sun, Jinlin Hou, Hongkai Wu, Zhanhui Wang

**Affiliations:** ^1^State Key Laboratory of Organ Failure Research, Guangdong Provincial Key Laboratory of Viral Hepatitis Research, Department of Infectious Diseases and Hepatology Unit, Nanfang Hospital, Southern Medical University, Guangzhou, China; ^2^Department of Infectious Diseases, The First Hospital of Jiaxing, Jiaxing, China; ^3^State Key Laboratory of Respiratory Disease, The First Affiliated Hospital of Guangzhou Medical University, Guangzhou Medical University, Guangzhou, China

**Keywords:** high-throughput sequencing, chronic hepatitis B infection, nucleos(t)ide analogs, hepatitis B e antigen seroconversion, T cell receptor

## Abstract

Long-term treatment with nucleos(t)ide analogs (NUCs) can improve the antiviral T cell response in chronic hepatitis B (CHB) patients. Whether and to what extent the T cell response is improved by NUCs in the early stage leading to hepatitis B e antigen (HBeAg) seroconversion remain to be clarified. A total of 22 CHB patients undergoing 2-year telbivudine-based therapy were enrolled, including 10 exhibiting a complete response (CR) and 12 exhibiting a non-complete response (NCR) according to HBeAg seroconversion at week 52. Peripheral CD4^+^ and CD8^+^ T cells were sorted at baseline, weeks 12, and 24. The T cell receptor β chain (TCRβ) complementarity-determining region 3 was analyzed by unbiased high-throughput sequencing. Compared with NCR group, patients in CR group had a much lower percentage of persistent clonotypes (*P* < 0.001) but remarkably higher percentages of new and expanded clonotypes (*P* < 0.05) between any two time points for both CD4 and CD8 subsets. The CD4 T cells exhibited a stronger response than CD8 population in the patients. The number of new and expanded clonotypes was inversely associated with the decline of viral antigen. In conclusion, NUC-based therapy induces a broad and vigorous T cell response with rapid decline of antigenemia during the early stage of treatment. A broad T cell expansion is crucial for HBeAg seroconversion. Our findings suggest that the potent suppression of hepatitis B virus replication by NUC monotherapy complemented with additional immunomodulatory strategies may increase the likelihood of a functional cure for CHB in the future.

## Introduction

The currently available options for chronic hepatitis B (CHB) treatment include nucleos(t)ide analogs (NUCs) and interferon-α (IFN-α). One of the goals of antiviral therapy in hepatitis B e antigen (HBeAg)-positive patients is achieving HBeAg seroconversion, an indicator of a more favorable outcome of the antiviral therapy (1). However, approximately only 20–30% of HBeAg-positive patients achieve HBeAg seroconversion during 2–3 years of continuous NUC treatment (2, 3). Many studies have defined the robust and multiepitope-specific CD4 and CD8 T cell responses that mediate the spontaneous clearance of an acute hepatitis B virus (HBV) infection (4–6), whereas weak or exhausted T cell function occurs during a chronic HBV infection, leading to persistent infection (7–10). Nevertheless, multiple studies have shown an increased frequency and improved function of HBV-specific CD4 and CD8 T cells in CHB patients after NUC therapy (11–19), although the restoration of functional T cells is transient and not vigorous enough to control HBV for a prolonged period (11).

The antigen specificity of a T cell is determined by the T cell receptor (TCR) expressed on its surface. Over 90% of TCRs are composed of α and β chains (20). The massive diversity of TCRs is due to the somatic rearrangement of various V-J gene segments of the α chain and V-D-J gene segments of the β chain (21). Furthermore, the variable addition and deletion of nucleotides at the junctions between the gene segments contributes to greater diversity of the TCR repertoire (22). Complementarity-determining region 3 (CDR3) in the T cell receptor β chain (TCRβ), which falls at the junction between the V-D-J gene segments, forms the center of the TCR antigen-binding site. Therefore, the molecular features of CDR3s can be used to monitor T cell response to antigens.

T cell receptor diversity is closely associated with the effective elimination of pathogens by the host immune system, treatment effectiveness and the outcome of multiple diseases (23–27). Several studies have investigated TCRs in HBV-infected patients. Skewed variable TCR patterns were found in different T cell subsets of patients with acute hepatitis B and during different clinical phases of CHB compared with healthy controls (28–33). In addition, several researchers have studied the relationship between TCRs and antiviral treatment outcomes in CHB patients (34, 35). However, all these studies assessed the TCR using conventional methods, such as quantitative PCR, CDR3 spectratyping, and Sanger sequencing, all of which yield extremely limited information, given that only the length or a small number of TCR CDR3 sequences can be determined.

The high-throughput sequencing of TCRs allows in-depth profiling of the T cell repertoire, thereby providing an unprecedented amount of information at the sequence level for any given sample. Additionally, the composition, distribution, and diversity of CDR3s can be elucidated and the relative levels quantified. To date, a very limited number of studies have investigated the TCR repertoire in CHB patients using high-throughput sequencing (36, 37), and only the usage of Vβ and Jβ gene segments was analyzed in these studies. Furthermore, while previous studies of NUC-treated patients have shown the restoration of T cell function by stimulating peripheral blood mononuclear cells (PBMCs) and staining for cytokines, the type and extent of antiviral T cell response that required to be restored to achieve control of a chronic HBV infection upon antiviral therapy remain unclear. In this study, we aimed to elucidate the characteristics and dynamic perturbations of the CD4 and CD8 TCR repertoires that were associated with a decline in HBV antigen in CHB patients treated with NUCs. We conducted a longitudinal study to quantify dynamic changes in the global CD4 and CD8 TCR CDR3 repertoires during an early stage of NUC therapy in CHB patients with or without HBeAg seroconversion. With an in-depth molecular analysis, our results provide a new quantitative insight into understanding T cell responses in CHB patients undergoing NUC treatment. Our data reveal the importance of a broad T cell expansion along with a decline in viral antigen to control HBV in CHB patients undergoing NUC therapy, especially during the first 12 weeks of treatment.

## Materials and Methods

### Patients

A total of 22 HBeAg-positive CHB patients were enrolled from Nanfang Hospital (Guangzhou, China). All the patients participated in a prospective clinical trial of telbivudine-based therapy (38, 39). The inclusion criteria were as follows: hepatitis B surface antigen (HBsAg)-positive status for at least 6 months; HBeAg-positive and anti-HBe-negative; HBV DNA >5 log_10_ copies/mL; alanine aminotransferase (ALT) ≥2 and <10× the upper normal limit; and without any antiviral treatment within 6 or 12 months. The exclusion criteria have been described elsewhere (38). The 22 patients were subdivided into complete response (CR, *n* = 10) and non-complete response (NCR, *n* = 12) groups based on HBeAg status and HBV DNA level at weeks 52 and 104 of treatment. Patients in the CR group achieved HBeAg seroconversion and had a level of serum HBV DNA of <300 copies/mL at week 52, which was sustained at week 104, while patients in the NCR group maintained HBeAg positivity and an HBV DNA level >300 copies/mL at weeks 52 and 104. All the patients achieved normal ALT levels at weeks 52 and 104. The baseline characteristics of the patients are provided in Table 1. This study was conducted according to the Declaration of Helsinki and was approved by the Ethics Committee of Nanfang Hospital. Written informed consent was obtained from all the patients.

**Table 1:** Clinical characteristics of enrolled patients at baseline.

	CR (*N* = 10)	NCR (*N* = 12)	*P*-value
Gender (male/female)^b^	8/2	12/0	0.195
Age, years^a^	26 (21.75–27.25)	29 (23.5–35.25)	0.185
Hepatitis B virus (HBV) Genotype (B/C)^b^	7/3	8/4	1.000
ALT (ULN)^a^	2.95 (2.20–6.49)	3.28 (2.36–5.56)	0.947
HBV DNA, log_10_ copies/mL^a^	8.58 (6.92–9.08)	9.07 (8.62–9.56)	0.056
HBsAg, log_10_ IU/mL^a^	4.43 (3.86–4.56)	4.88 (4.49–5.06)	0.015
HBeAg, log_10_ PEIU/mL^a^	2.39 (1.61–3.34)	3.17 (2.85–3.31)	0.391
Anti-HBc, log_10_ IU/mL^a^	4.35 (4.21–4.51)	4.06 (3.92–4.26)	0.018

*^a^Median (25–75% percentile), Mann–Whitney *U* test*.

*^b^Fisher’s exact test*.

*CR, complete response, patients with HBeAg seroconversion at week 52 and 104; NCR, non-complete response; patients without HBeAg seroconversion at week 52 or 104; ALT, alanine aminotransferase; HBsAg, hepatitis B surface antigen; HBeAg, hepatitis B e antigen; anti-HBc, hepatitis B core antibody; ULN, upper limit of normal*.

### Assays for Serological Markers and HBV DNA

Serological markers for HBV were detected using ARCHITECT i2000SR (Abbott Laboratories, Chicago, IL, USA). The HBV DNA level was quantified using the Roche COBAS Taqman platform (with a lower limit of detection of 12 IU/mL or 69.84 copies/mL). Serum ALT levels were assessed at local laboratories according to standard procedures. HBV genotypes were identified by S gene sequencing.

### Isolation of PBMCs and Cell Sorting

Peripheral blood samples were collected at baseline and at weeks 12 and 24 of treatment. PBMCs were isolated by density-gradient centrifugation according to standard protocols and were cryopreserved in liquid nitrogen until analysis. For cell sorting, the PBMCs (0.1–1 × 10^7^) were thawed, washed with RPMI-1640 containing 10–20% FBS, and then treated with 100 U/mL DNase I (Roche) to degrade DNA released from dead or dying cells in order to reduce cell clumps. The remaining PBMCs were labeled with Live/Dead (Life Technologies), CD4-APC, CD8-FITC, and CD19-PE (BD Biosciences) and were sorted using a FACSAria III Cell Sorter (BD Biosciences). The staining profile and gating strategy for cell sorting are shown in Figure S1 in Supplementary Material.

### RNA Preparation and TCRβ Deep Sequencing

Total RNA was extracted from the sorted lymphocytes using TRIzol reagent (Invitrogen). Unbiased TCRβ libraries were prepared using 5′ rapid amplification of cDNA ends strategy, as previously described (40), followed by Illumina HiSeq sequencing.

### TCRβ Sequence Analysis

T cell receptor β chain V, D, and J gene identification, CDR3 sequence extraction and error corrections in clean reads were performed using MiXCR (41). The productive TCRβ sequence reads were filtered by removing: (i) any read with CDR3s shorter than 4 amino acids; (ii) CDR3 contigs with a length that was not a multiple of 3; and (iii) contigs containing stop codons. TCR diversity was evaluated by Shannon entropy (42). A method for the analysis of dynamic changes in the T cell repertoire has been previously described (26) and was used with some modifications. First, all clonotypes (i.e., unique amino acid TCRβ sequences) were assigned to four classes according to their log_10_-transformed frequencies as follows: 1+ referred to clonotypes in the lowest log_10_ frequency range; 2+ denoted clonotypes occupying 95% of the total repertoire and excluded clonotypes in 1+; 4+ referred to the most abundant 100 clonotypes; and 3+ referred to the remaining clonotypes that were not classified as 1+, 2+, or 4+. The cumulative frequency of each clonotype class (i.e., the clone size of each individual clonotype) was analyzed by calculating the sum of the same class of clonotypes at the indicated time point. Finally, each clonotype was tracked over the treatment time points and sorted into one of five categories: ablated (clones not present at later time points); depleted (clones downgraded to lower frequency classes); persistent (clones that were stable in the same class between time points); expanded (clones upgraded from a lower to a higher frequency class); or new (clones not present at prior time points).

### Phylogenetic Analysis

Phylogenetic tree was constructed according to the neighbor-joining method using Mega 7.0 (43). Bootstrap support was determined by 500 resamplings of the sequences.

### Statistical Analysis

The data were expressed as medians. The Mann–Whitney *U*-test and Fisher’s exact test were used for two-group comparisons. The Friedman’s *M* test was used to compare values among three time points during treatment. The q test was subsequently used to determine which two values were significantly different if the *P*-value was < 0.05 for Friedman’s *M* test. The Spearman’s rank order correlation coefficient was used to calculate correlations. Two-sided *P*-values < 0.05 were considered statistically significant.

## Results

### Patient Characteristics

At baseline, there were no significant differences in age, gender, genotype, or the levels of HBeAg or HBV DNA between the CR and NCR patients; differences were observed in HBsAg and hepatitis B core antibody levels between the groups (Table 1). The dynamic changes in HBV serological markers are shown in Figure S2 in Supplementary Material. No patients achieved HBsAg loss at week 104. The number of sorted cells in each T cell subset was also comparable between the two groups of patients at each time point (Table S1 in Supplementary Material).

### Patient TCRβ Repertoire Profiles

A total of 229,756,079 and 153,420,480 clean reads were obtained from CD4 and CD8 T cells, respectively, by high-throughput sequencing. After filtering the data with MiXCR, we obtained 194,195,801 and 119,574,667 productive TCRβ reads from CD4 and CD8 T cells, respectively, which were assembled for a total of 27,228 and 25,035 unique CDR3 nucleotide (nt) clonotypes encoding 25,840 and 23,393 unique CDR3 amino acid (aa) clonotypes per sample, respectively. Details of the TCR sequences are displayed in Table S1 in Supplementary Material. Both the clean reads and total unique TCRβ reads at each of the three time points were comparable between the CR and NCR groups in each cell subset.

### The Usage of Vβ and Jβ Gene Segments

**Figure 1:**
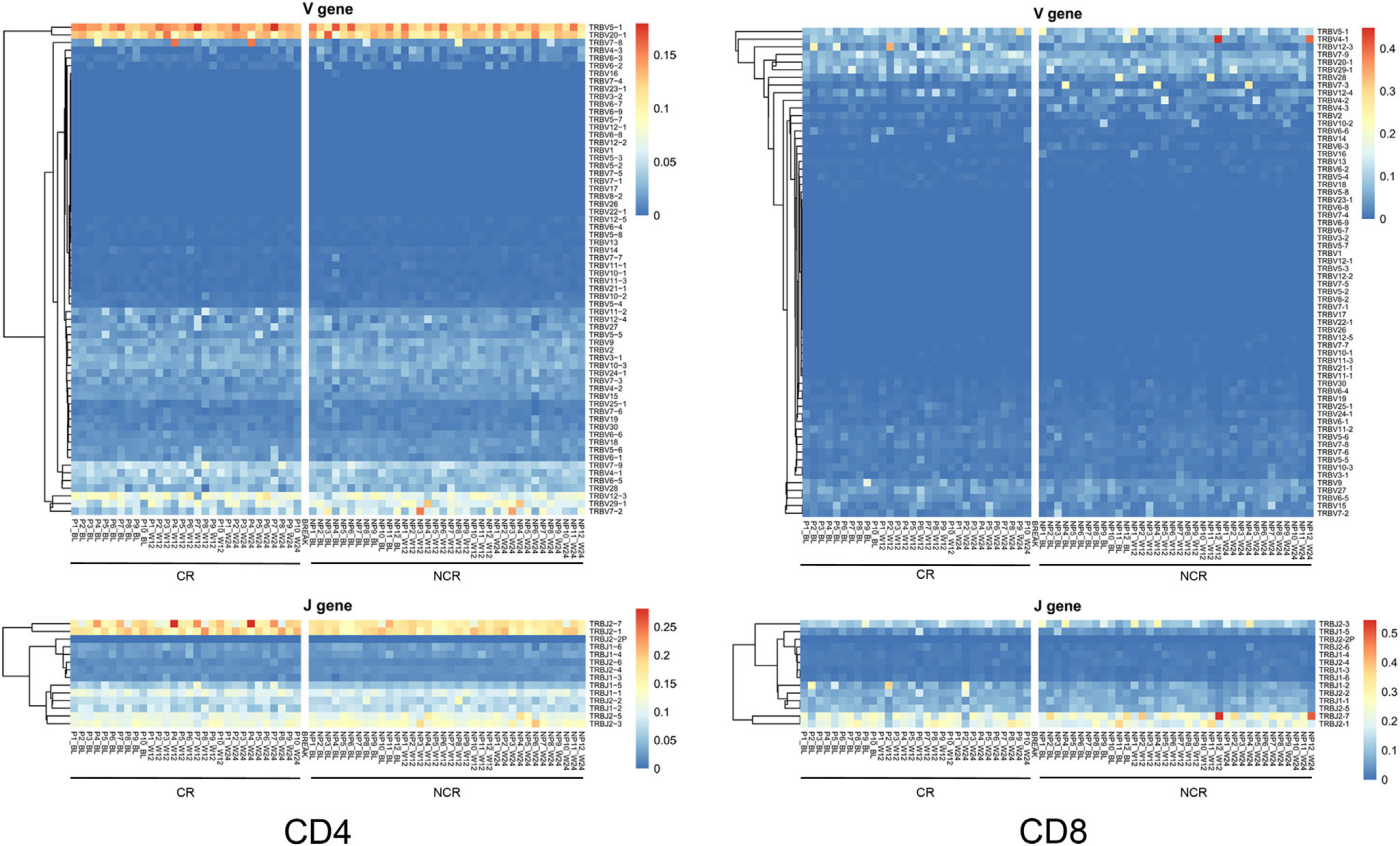
Heatmap of Vβ and Jβ gene segments usage in CD4 and CD8 T cells in the complete response (CR) and non-complete response (NCR) patient groups. The heatmap bar indicates the usage frequency of the Vβ or Jβ gene segments in each sample. The sample numbers of the CR and NCR groups are indicated on the *x*-axis.

In the CD4 T cells, 64 Vβ and 14 Jβ gene segments as well as 790 VJ and 2,266 VDJ gene combinations were identified. In CD8 T cells, 64 Vβ, 14 Jβ, 795 VJ, and 2,266 VDJ gene combinations were identified. The usage patterns of Vβ and Jβ gene segments in each sample at each time point are shown in Figure 1. No obvious preferential usage of Vβ and Jβ gene segments was observed between the CR and NCR groups in either the CD4 or CD8 subset. Nevertheless, we found two to eight Vβ segments with significantly different expression profiles between the CR and NCR groups in each T cell subset at each time point (Figure S3 in Supplementary Material). The Vβ gene segments in CD4 T cells clustered nearly distinctively between the CR and NCR groups based on principal component analysis; however, no similar phenomenon was observed in CD8 T cells (Figures S4A,B in Supplementary Material).

### Differing Diversity but Similar Patterns of Change in CD4 and CD8 T Cells in CR and NCR Patients

**Figure 2:**
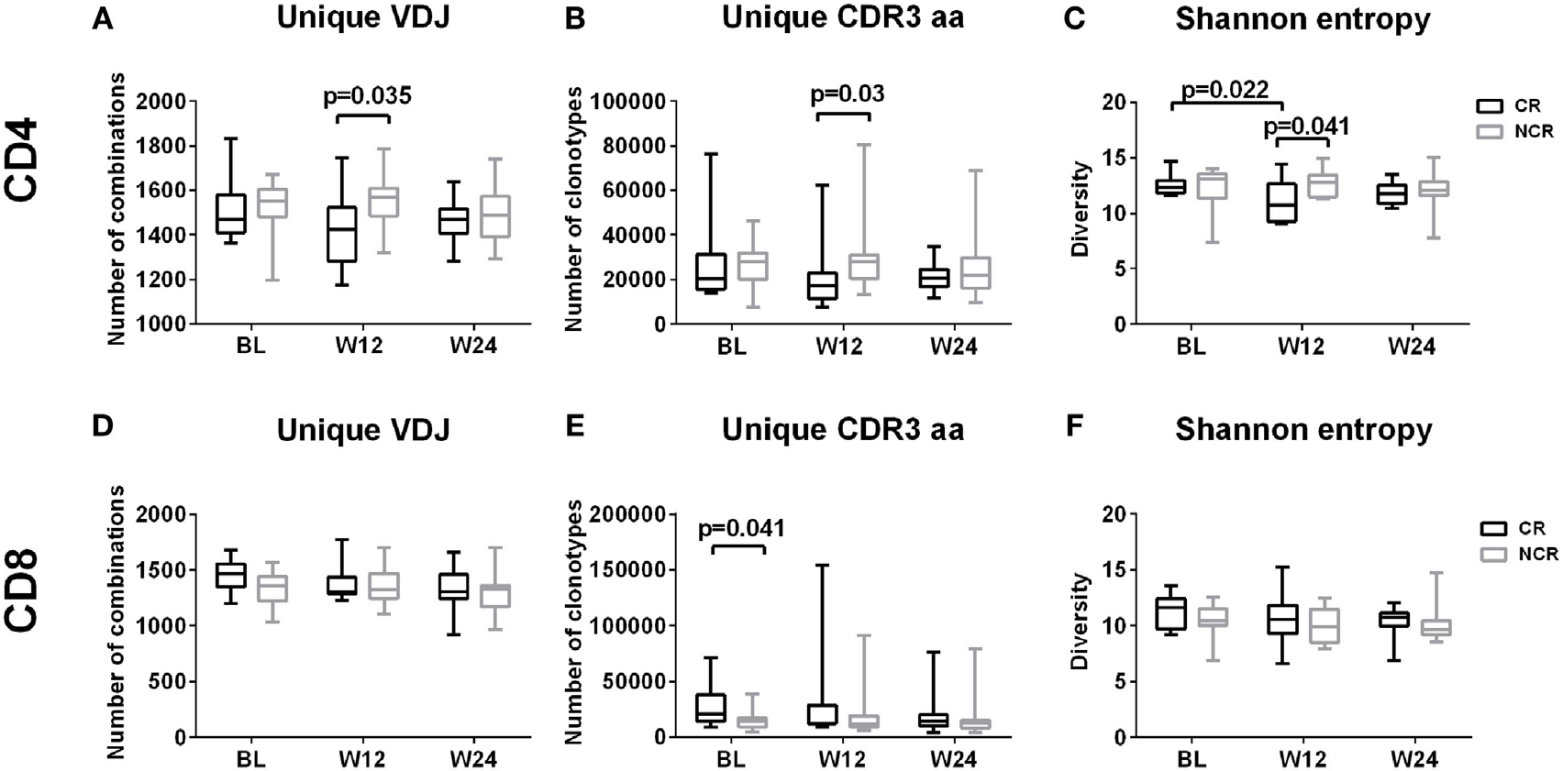
Diversity of CD4 and CD8 T cell repertoires during early nucleos(t)ide analog treatment. Longitudinal analysis of the number of unique VDJ combinations **(A,D)**, complementarity-determining region 3 (CDR3) aa clonotypes **(B,E)**, and Shannon entropy values **(C,F)** in CD4 and CD8 T cells in the complete response (CR) and non-complete response (NCR) groups, respectively; *P*-values less than 0.05 are shown. The *x*-axis represents treatment timepoints. BL, baseline; W12, week 12; W24, week 24.

The numbers of unique VDJ combinations, aa clonotypes, as well as the Shannon entropy values, were relatively lower in CD4 subset (Figures 2A–C) but were higher in CD8 subset (Figures 2D–F) in the CR patients at baseline, week 12 and week 24 of treatment, although statistically significant differences were observed only at week 12 for CD4 cells and at baseline for CD8 cells. This result indicates that there was a relatively higher diversity in CD8 T cells, while the CD4 cells exhibited reduced diversity in CR patients compared with the NCR patients. Compared with NCR group, the relatively lower level of diversity in CD4 cells in the CR patients at baseline reached significance at week 12, as indicated by the VDJ combinations (1,423 vs 1,570; *P* = 0.035), aa clonotypes (17,255 vs 27,840; *P* = 0.03), and Shannon entropy values (10.80 vs 12.78; *P* = 0.041). The diversity of CD4 TCRβ repertoire significantly decreased from baseline to week 12 during treatment in the CR patients (12.30 vs 10.80; *P* = 0.022; Figure 2C). However, the significantly higher number of aa clonotypes (20,710 vs 14,600; *P* = 0.041) in CD8 cells of the CR patients at baseline were comparable to those in the NCR patients at week 12. In accord with this, the HBV DNA, HBsAg, and HBeAg levels declined rapidly during the first 12 weeks of treatment compared with the other time points (Figure S2 in Supplementary Material). Collectively, these data suggest a trend that the diversity of CD4 and CD8 TCRβ repertoires were changed during the early stage of NUC therapy along with the rapid decline of viral antigen levels compared with the NCR patients, although a significant difference was only observed in CD4 T cells.

### Clone Distribution of the CD4 and CD8 TCR Repertoires

**Figure 3:**
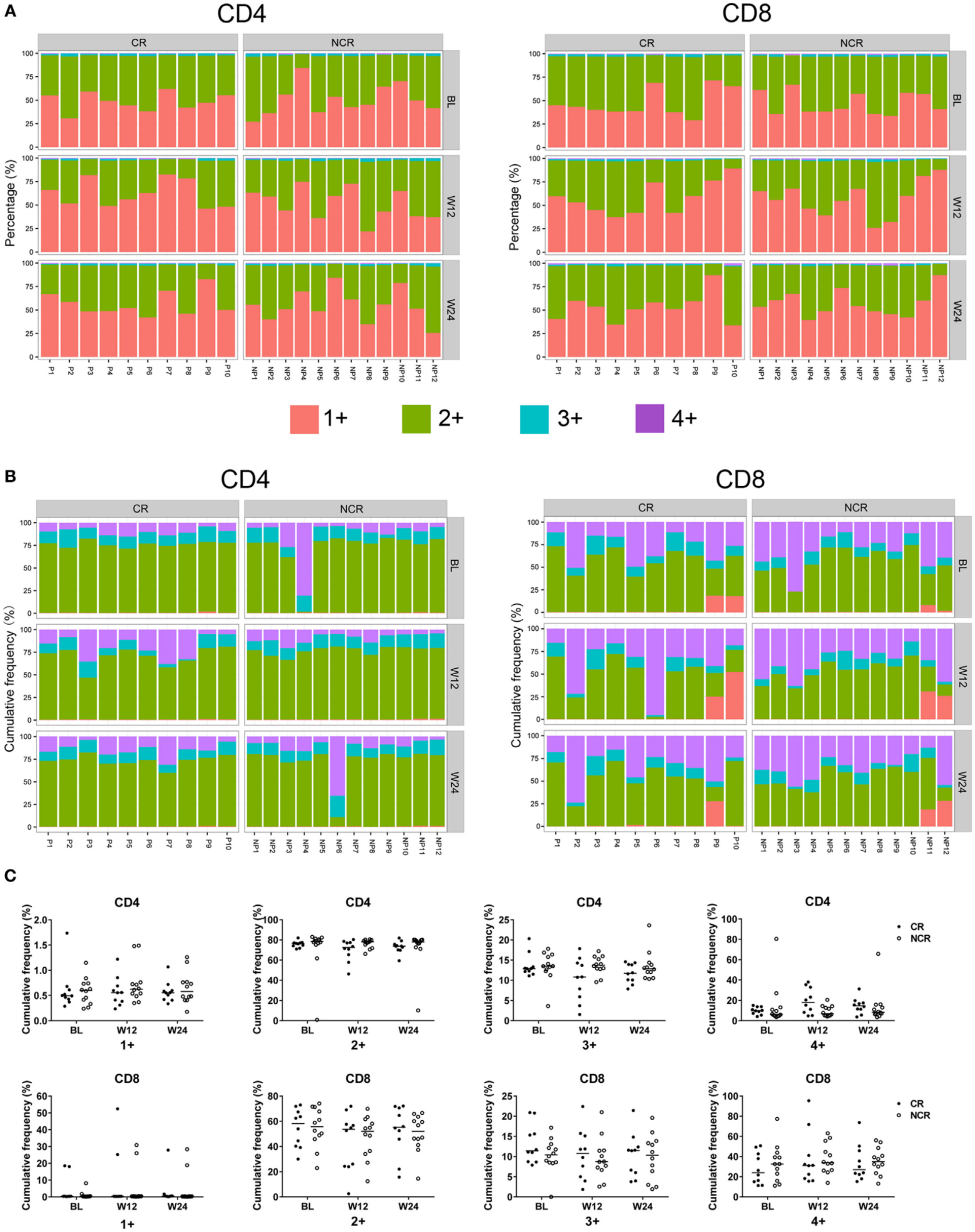
Clone distribution of CD4 and CD8 T cells in the chronic hepatitis B patients at baseline, week 12, and week 24 of treatment. Each CD4 and CD8 clonotype was assigned to one of four classes at each time point based on the frequency of the clonotype, and the percentage of unique clonotypes **(A)** and the cumulative frequency of clonotypes **(B)** within identical classes are shown. **(C)** Comparisons of the cumulative frequency of CD4 and CD8 clonotypes in the four classes at each time point. No significant differences were observed. The patient numbers are indicated on the *x*-axis. P, patients in complete response (CR) group; NP, patients in non-complete response (NCR) group.

To determine how the TCRβ repertoires of CD4^+^ and CD8^+^ T cells changed with NUC treatment, we first tracked the dynamic changes in all clones at baseline, weeks 12, and 24. The individual clones in both CD4 and CD8 cells exhibited remarkable changes in all the patients (Figure S5 in Supplementary Material). When changes in the dynamic frequency of the most highly abundant clones (the top 5, 10, 20, and 50) were analyzed separately, some T cells showed a persistent increase, while others exhibited an increase followed by a decrease or *vice versa* (Figure S6 in Supplementary Material). We further analyzed the dynamic changes in each clonotypic frequency by stratifying all the clonotypes into four classes according to their frequencies (details are provided in the Section “Materials and Methods”). In all the patients, the vast majority of T cell clones belonged to class 1+ or 2+ (Figure 3A). The 100 most abundant clonotypes in CD8 T cells accounted for a greater proportion of the TCRβ repertoire compared with those present in CD4 cells, suggesting greater expansion of the most abundant clones in CD8 T cells (Figure 3B). Nevertheless, the CR and NCR groups had similar cumulative frequencies across all four classes at each time point for both cell subsets (Figure 3C).

### CR Patients Were Characterized by Greater Perturbations of Both CD4 and CD8 TCR Repertoires

**Figure 4:**
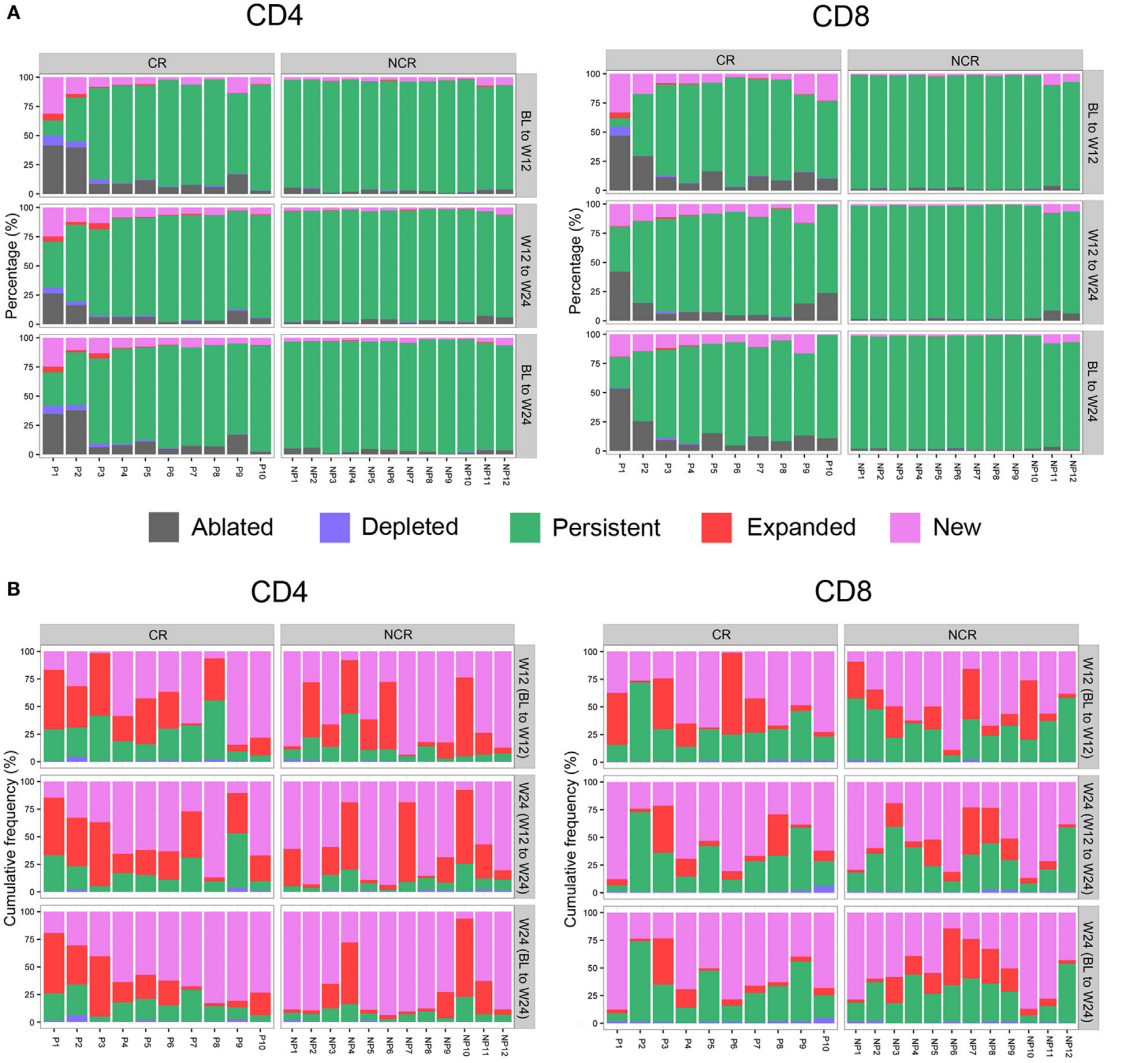

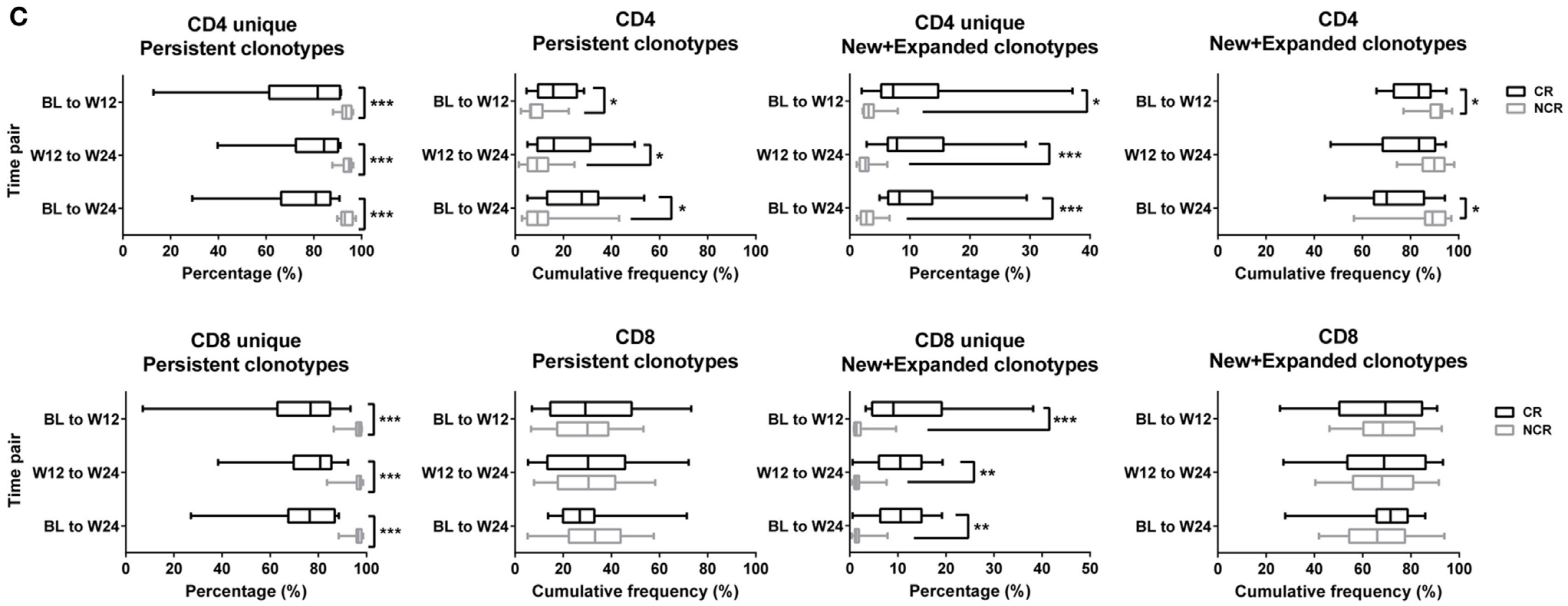
Longitudinal evaluation of T cell clonotypes during early nucleos(t)ide analog treatment. **(A)** Percentage of unique CD4 and CD8 clonotypes in five categories between baseline (BL) to week 12 (W12), week 12 (W12) to week 24 (W24), and BL to week 24 (W24). **(B)** The cumulative frequency of CD4 and CD8 clonotypes in the four categories (depleted, persistent, expanded, and new) between the three time pairs was assessed by calculating the sum of the frequency in each category at the later time point, for example, W12 (BL to W12), W24 (W12 to W24), or W24 (BL to W24). **(C)** Comparisons of CD4 and CD8 clonotypes in different categories between each time pair; ***P < 0.001; **P < 0.01; *P < 0.05. The patient numbers are indicated on the *x*-axis. P, patients in complete response (CR) group; NP, patients in non-complete response (NCR) group.

To further determine how the frequency of CD4 and CD8 TCRβ repertoires changed, all the clones in classes 1+ through 4+ in each patient were assigned to one of five categories—ablated, depleted, persistent, expanded, or new—according to the criteria described in the Section “Materials and Methods.” The percentage of persistent unique clonotypes was the highest compared with that of the other four categories between any two time points in either subset (Figure 4A). However, the median cumulative frequencies of persistent clonotypes in CD4 and CD8 cells were only 10.68 and 28.84%, respectively (Figure 4B). The cumulative frequency of new and expanded clonotypes in CD4 was higher than that in CD8 subset in the patients (Figure 4B). The new and expanded clonotypes, which were 4.05 and 3.65% of the total CD4 and CD8 clonotypes, respectively, constituted 87.76 and 69.90% of the CD4 and CD8 repertoires, respectively (Figures 4A,B).

We next compared the percentage and cumulative frequency of each category of T cell clone between the CR and NCR patients. The percentage of persistent clonotypes was lower in CR than in the NCR group between any two time points in both CD4 and CD8 subsets (all *P* < 0.001; Figures 4A,C). In contrast, patients in the CR group had much higher combined percentages of new and expanded clonotypes than those in the NCR group between any two time points in both CD4 and CD8 cells (all *P* < 0.05; Figures 4A,C). The number of ablated clonotypes in CD4 T cells at week 12 was much higher than that of newly appearing clonotypes compared with baseline (*P* = 0.021), leading to a lower diversity of CD4 cells at week 12 in CR group. When clone size was considered, the CR group exhibited a higher cumulative frequency of persistent clones (all *P* < 0.05) and a lower combined cumulative frequency of new and expanded clonotypes in CD4 T cells during the first 12 weeks of treatment (70.14 vs 89.11%; *P* = 0.021), while no difference was observed in the cumulative frequency of any category clones among the CD8 T cells (Figure 4C).

Taken together, these results indicate that a turnover of the TCRβ repertoire occurred in both CD4^+^ and CD8^+^ T cells of the CHB patients undergoing NUC treatment. Patients with a CR underwent more profound perturbations and broader clonal expansions of both the CD4 and CD8 T cell repertoires, but the clone size of each new and expanded clonotype was smaller.

### Correlation between the Decline of HBV Antigens and T Cell Expansion

With the rapid decline in HBV DNA, HBeAg, and HBsAg levels from baseline to week 12, a correlation between viral antigen and T cell expansion was observed for both CD4 and CD8 T cells. The percentage of unique new and expanded clonotypes exhibited a negative correlation, while persistent clonotypes were positively correlated with antigen levels at week 12. In addition, the amount of HBeAg reduction from baseline to week 12 was consistently and significantly associated with the percentage of new and expanded clonotypes (Table 2). These results suggest the importance of viral antigen suppression in inducing a strong T cell response.

**Table 2:** Correlation between hepatitis B virus (HBV) antigen levels and the percentage of clonotypes.

Subject	Time point/time pair	Correlation coefficient	Persistent (BL to W12)		New + expanded (BL to W12)	
			CD4	CD8	CD4	CD8
Hepatitis B surface antigen (log_10_ IU/mL)	W12	*r*	0.458	0.548	−0.478	−0.509
		*P* value	0.037	0.01	0.028	0.018

	BL to W12	*r*	−0.155	−0.24	0.292	0.288
		*P* value	0.504	0.294	0.199	0.205

Hepatitis B e antigen (log_10_ PEIU/mL)	W12	*r*	0.524	0.560	−0.653	−0.522
		*P* value	0.012	0.007	0.001	0.013

	BL to W12	*r*	−0.575	−0.621	0.542	0.582
		*P* value	0.005	0.002	0.009	0.004

HBV DNA (log_10_ copies/mL)	W12	*r*	0.552	0.591	−0.419	−0.463
		*P* value	0.008	0.004	0.053	0.03

	BL to W12	*r*	−0.226	−0.252	0.045	0.117
		*P* value	0.311	0.257	0.844	0.604

*Spearman correlation was analyzed for samples of baseline and week 12 in total 22 patients; W12, the level of indicated marker at week 12 of treatment; BL to W12, the decline range of given HBV markers from baseline to week 12, calculating as the titer of a given marker at baseline minus that at week 12; r, correlation coefficient; persistent, percentage of unique persistent clonotypes; new + expanded, combined percentage of unique new and expanded clonotypes*.

### CDR3 aa Sequence of the Top 1 Persistent Clonotype in CD8 Cells Differentiates the CR and NCR Groups

**Figure 5:**
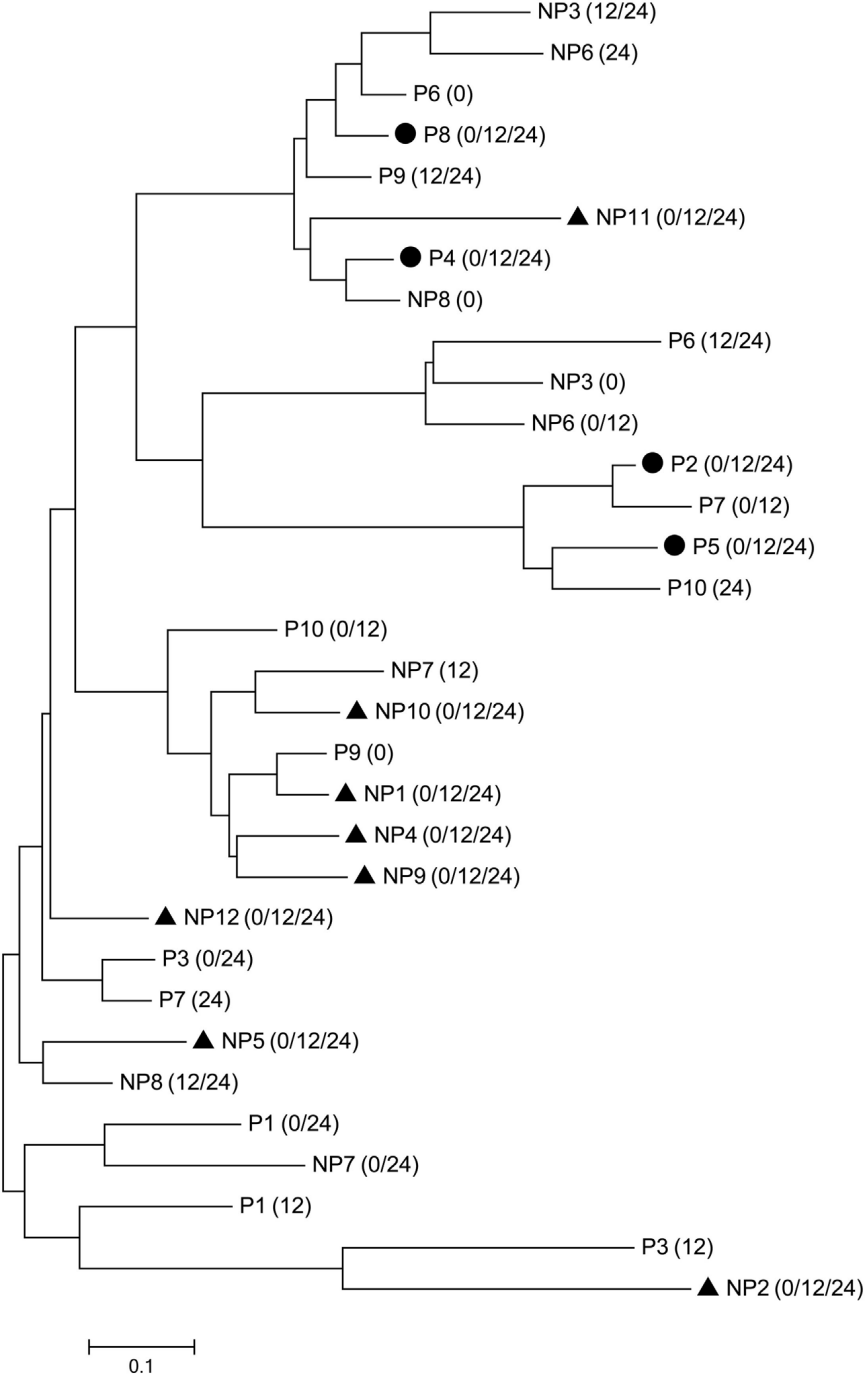
Clustering of the top 1 CD8 clonotypes at each time point. The top 1 complementarity-determining region 3 (CDR3) sequences of the complete response (CR) and non-complete response (NCR) groups are denoted by P (CR group) or NP (NCR group) + patient ID, respectively. The time point of the CDR3 sequence is indicated in parentheses. The circle (●) and triangle (▲) represent identical CDR3 sequences at baseline, week 12, and week 24 within individual patients from the CR and NCR groups, respectively.

We next investigated whether the CR and NCR groups could be distinguished based on the CDR3 aa sequences. The CDR3 aa sequences of the most dominant clonotype (top 1) in each sample were listed separately according to CD4 or CD8. Four CR and eight NCR patients showed identical top 1 CD8 CDR3 aa sequence at all three time points in personally, while the other patients had different top 1 clonotypes for at least one time point. Interestingly, when all the top 1 CDR3 aa sequences were phylogenetically analyzed, the four patients in the CR group clustered separately from seven of the eight NCR patients (Figure 5). However, no similar phenomenon was observed in CD4 subset (data not shown). These findings suggest that the persistent top 1 CDR3 aa sequences in CD8 clonotypes were discrepant between two groups and may distinguish patients with different outcome of NUC-based treatment.

## Discussion

T cells play crucial roles in HBV infection, with vigorous and multiepitope-specific CD4 and CD8 T cell responses observed in patients with acute infection, while exhausted T-cell activity is found in cases of chronic HBV infection. The goal of CHB treatment with NUCs or IFN-α is to suppress HBV replication and to reduce or prevent the progression of liver disease. Accumulating evidence suggests that NUC treatment improves the antiviral T cell function in CHB patients along with the decline of antigenemia, especially in patients with HBsAg loss. Traditionally, HBV-specific T cells have been measured by stimulating PBMCs with HBV peptide pools and staining cytokines. However, the characteristics of T cell repertoires, including clonal composition, diversity at a sequence-level resolution, and dynamic changes, have rarely been analyzed. Therefore, what kind and to what extent of antiviral T cell response is required to achieve control of chronic HBV infection upon antiviral therapy remains unclear. Accordingly, with the unbiased amplification of TCR sequences combined with high-throughput sequencing, we longitudinally investigated the dynamic changes of peripheral global CD4 and CD8 TCR repertoires at baseline, week 12 and week 24 in NUC-treated CHB patients with or without HBeAg seroconversion at weeks 52 and 104. The dynamic changes in all the T cell frequencies and numbers of clonotypes were calculated based on CDR3 sequences, allowing the quantity and quality of the T cell response by antiviral therapy to be assessed.

One important finding of this study concerns the heterogeneous dynamic change in individual clonotypes. By tracking CD4 and CD8 clonotypes during the first 24 weeks of antiviral therapy, we observed that the percentage of changeable clonotypes was significantly higher in CR than in the NCR patients, especially during the first 12 weeks of treatment. A large number of new and expanded clones were identified in both CD4 and CD8 T cells in the CR patients coinciding with a rapid decline in antigenemia and HBV DNA at week 12. In contrast, both the CD4 and CD8 TCR repertoires in the NCR group had a higher percentage of persistent clonotypes compared with the CR group. These data indicate a broader clonotypic T cell expansion and vigorous T cell responses of CD4 and CD8 in the CR patients compared with those in NCR patients. Consistent with this result, two important recent studies also revealed that patients with HBsAg loss upon antiviral therapy presented a broad and strong *in vitro* HBV-specific T cell response as assessed by analyzing cytokine production (19, 44). Using high-throughput sequencing and the calculation of global TCR CDR3 repertoires in PBMCs with no *in vitro* manipulation such as cell culture or stimulation, we quantitatively demonstrated at a great depth a robust T cell expansion in patients with HBeAg seroconversion. However, when the clone size was considered, the combined cumulative frequencies of new and expanded clonotypes in the CR group was comparable in the CD8 subset and significantly lower in the CD4 subset compared with those in the NCR group, indicating that the size of each new and expanded clonotype was restricted. The higher percentage and lower cumulative frequency of the new and expanded clonotypes in the CR patients suggest that a higher-quality and broader T cell expansion against multiple epitopes is more important than a larger clone size with a narrower T cell response for HBeAg seroconversion in CHB patients undergoing NUC therapy. Our data also demonstrate the correlation of antigen decline and clone expansion, indicating the crucial role of HBV suppression in functional T cell responses.

In the current study, we noticed that the cumulative frequency of new and expanded clonotypes was significantly higher in CD4 than in CD8 subset in the patients. We also observed differences in the patterns of change in the clonotypic composition and diversity of CD4 TCR repertoires between HBeAg seroconverters and non-seroconverters. The CD4 T cells in the CR group exhibited fewer CDR3 clonotypes in parallel with a less diverse repertoire at week 12 of treatment compared with those in the NCR group. This result is due to a greater clonal expansion of the CD4 cells in the CR patients after 12 weeks of NUC-based treatment, which replaced the ablated clones. These data suggest a more vigorous CD4 response in CR compared to the NCR patients upon NUC treatment. Recent studies have also indicated that CD4-mediated responses are significantly stronger than CD8 responses in NUC-treated patients with HBsAg loss (19). Consistent with our result, higher level of serum IL-21 (45) and an increased frequency of circulating CXCR5^+^CD4^+^ T cells during the first 12 weeks of NUC treatment (46) were reported to be associated with early HBeAg seroconversion at week 52 in CHB patients. Similarly, Chen et al. also revealed that effective-treated patients had an increased frequency of peripheral blood CD4^+^ T cells at week 12 upon telbivudine therapy (15). Collectively, a broad and extensive clonal expansion of CD4^+^ T cells during the first 12 weeks of NUC treatment is essential to antiviral treatment outcomes of CHB patients.

It is well known that viral clearance is mediated by both cytolytic and non-cytolytic functions of the CD8 T cell response with the help of CD4 cells; furthermore, improved function and expansion of CD8^+^ T cells during NUC treatment is related to a favorable virological outcome (34). In addition to the broad new and expanded T cell responses, we devoted particular attention to highly abundant clonotypes among the CD8 cells. Patients in the CR group had a higher diversity of top CD8 clonotypes than did the NCR group (data not shown), indicating that a more even distribution of top clonotypes is related to HBeAg seroconversion, while oligoclonal expansion against limited antigen epitopes is likely linked to the absence of HBeAg seroconversion. Notably, based on a phylogenetic analysis, the CDR3 aa sequences of the top 1 persistent CD8 clonotypes clustered separately for the CR and NCR patients. Whether the top 1 clonotype is HBV-specific or could be used to predict the treatment outcome requires additional study.

Because a large number of new and expanded clones were identified in this study, we were unable to confirm whether these T cells were HBV-specific. Previous studies have indicated that the frequency of HBV-specific T cells is very low in the PBMCs of CHB patients (47). Additionally, the exact phenotypic markers of HBV-specific T cells remain to be determined. This lack of information increases the difficulty of profiling HBV-specific T cell repertoires through cell sorting and high-throughput sequencing. Furthermore, studies have shown that the restoration of functional T cells induced by NUC therapy in CHB patients is transient. Therefore, alternative strategies are required to increase the frequency of HBV-specific T cells associated with NUC therapy. One potential strategy is the adoptive transfer of engineered HBV-specific TCR-redirected autologous T cells to improve the immune response (48).

In conclusion, by analyzing the clonotypic composition and diversity of peripheral CD4 and CD8 TCR repertoires and tracking dynamic changes in the different clonotypes in CHB patients upon NUC treatment, we have shown that substantial clonal expansions of the CD4 and CD8 T cell repertoires are associated with early treatment-induced HBeAg seroconversion. A stronger T cell response is closely associated with a rapid decline in viral antigen. Therefore, the robust inhibition of HBV replication complemented by immunomodulatory strategies in NUC monotherapy may improve the restoration of functional T cells and achieve complete HBV control.

## Ethics Statement

This study was conducted according to the Declaration of Helsinki, and was approved by the Ethics Committee of Nanfang Hospital. Written informed consent was obtained from all the patients.

## Author Contributions

ZW, JH, and YX conceived and designed experiments. YX, MZ, YL, MG, YC and CX performed the experiments. MZ, YX, MG, HD, XL and JS collected data. HW, YX and YL analyzed the sequencing data. ZW, YX and HW interpreted the data. ZW and YX wrote the manuscript. ZW, JH and JS approved the final manuscript. All authors had full access to the final version of the report and agreed to the submission.

## Conflict of Interest Statement

The authors declare that the research was conducted in the absence of any commercial or financial relationships that could be construed as a potential conflict of interest.

## Supplementary Material

Figure S1 Flow cytometric cell-sorting strategy for isolation of CD4^+^, CD8^+^ T cells and CD19^+^ cells. CD4^+^, CD8^+^ T cells and CD19^+^ cells were identified by gating on lymphocytes **(A)**, followed by exclusion of dead cells **(B)** and selection of CD4^+^, CD8^+^ T cells **(C)** and CD19^+^ cells **(D)**. The figures show representative FACS profiles of one chronic hepatitis B (CHB) patients and the strategy used for sorting.

Figure S2 The clinical information of enrolled patients during the 2-year of NUC-based treatment. Black dashed line, complete response (CR) group; black line, non-complete response (NCR) group. ALT, alanine aminotransferase; HBsAg, hepatitis B surface antigen; HBeAg, hepatitis B e antigen; Anti-HBc, hepatitis B core antibody.

Figure S3 Comparisons of the Vβ segments usage in peripheral CD4 and CD8 T cells in the complete response (CR) and non-complete response (NCR) patient groups during early NUC treatment. Different usage of the Vβ gene segments in CD4 **(A)** and CD8 **(B)** T cells in the CR and NCR patient groups (all *P*-values < 0.05, Mann–Whitney *U* tests). The fold change is calculated as the frequency of indicated Vβ gene segment in CR group divided by the frequency of corresponding Vβ gene segment in the NCR group at the same time point. BL, baseline; W12, week 12; W24, week 24.

Figure S4 Principal component analysis of Vβ gene segment usage in CD4 and CD8 T cells. Principal component analysis shows the usage patterns of Vβ gene segments of CD4 **(A)** and CD8 **(B)** samples of complete response (CR) group patients (red dots) and non-complete response (NCR) group patients (blue dots). PC1 refers to the first principal component, whereas PC2 indicates the second principal component.

Figure S5 Fluctuations of overall CD4 and CD8 clone repertoires in different groups. Longitudinal data points from each clonotype in each patient are connected by a line (frequency increased, red line; frequency decreased, blue line, frequency constant, green line). The log10-transformed frequency is indicated on the *y*-axis. The *x*-axis represents treatment timepoints. BL, baseline; W12, week 12; W24, week 24.

Figure S6 Tracking the dominant clones over the treatment. The top5 **(A,B)**, top 10 **(C,D)**, top 20 **(E,F)**, top 50 **(G,H)** clonotypes in CD4 (left) and CD8 (right) subsets in each patient [complete response (CR) group, upper lane; non-complete response (NCR) group, lower lane] are listed, respectively, and tracking them according to their frequency (*y*-axis) at baseline, week 12, and 24 (*x*-axis). Identical clonotypes are connected by a line (frequency increased, red line; frequency decreased, green line).

## Abbreviations

ALT, alanine aminotransferase; CDR3, complementarity-determining region 3; CHB, chronic hepatitis B; CR, complete response; HBeAg, hepatitis B e antigen; HBV, hepatitis B virus; NCR, non-complete response; NUCs, nucleos(t)ide analogs; PBMCs, peripheral blood mononuclear cells; TCRβ, T cell receptor β chain; IFN, interferon.
