# Supplementary material for: Dynamic Perturbations of CD4 and CD8 T Cell Receptor Repertoires in Chronic Hepatitis B Patients upon Oral Antiviral Therapy
Source: Front Immunol. 2017 Sep 14;8:1142. doi: 10.3389/fimmu.2017.01142 (PMC5603711; doi:10.3389/fimmu.2017.01142)
Supplement: Supplementary file 1 [file Table_1.DOCX]

| Table S1 Summary of TCRβ sequencing | | | | | | | | | | | | | |
| --- | --- | --- | --- | --- | --- | --- | --- | --- | --- | --- | --- | --- | --- |
| Patient | Group | Time point | Cell subset | Cell count (10^6^) | Clean reads | Total productive TCRβ reads | Productive TCRβ reads (%) | Unique Vβ gene segment | Unique Jβ gene segment | Unique VJ combination | Unique VDJ combination | Unique CDR3 nt clonotype | Unique CDR3 aa clonotype |
| P1 | CR | BL | CD4 | 2.70 | 2654013 | 2269660 | 85.52 | 58 | 13 | 555 | 1361 | 14567 | 14000 |
| P1 | CR | W12 | CD4 | 2.70 | 3057331 | 2570498 | 84.08 | 58 | 13 | 531 | 1286 | 12228 | 11895 |
| P1 | CR | W24 | CD4 | 2.50 | 2815180 | 2392481 | 84.99 | 55 | 13 | 543 | 1281 | 11817 | 11512 |
| P1 | CR | BL | CD8 | 3.20 | 3484118 | 2791279 | 80.11 | 59 | 13 | 580 | 1416 | 19352 | 18406 |
| P1 | CR | W12 | CD8 | 3.30 | 3527581 | 2836197 | 80.40 | 55 | 13 | 566 | 1360 | 15169 | 14597 |
| P1 | CR | W24 | CD8 | 2.20 | 2156409 | 1740725 | 80.72 | 56 | 13 | 512 | 1137 | 7063 | 6757 |
| P2 | CR | BL | CD4 | 7.00 | 2833582 | 2354188 | 83.08 | 63 | 13 | 631 | 1628 | 41498 | 38577 |
| P2 | CR | W12 | CD4 | 2.60 | 2881002 | 2407179 | 83.55 | 59 | 13 | 577 | 1485 | 20711 | 19963 |
| P2 | CR | W24 | CD4 | 4.20 | 2984919 | 2530093 | 84.76 | 53 | 13 | 558 | 1405 | 17401 | 16820 |
| P2 | CR | BL | CD8 | 4.20 | 2822904 | 2277004 | 80.66 | 62 | 13 | 586 | 1431 | 19944 | 19026 |
| P2 | CR | W12 | CD8 | 4.60 | 2757314 | 2199472 | 79.77 | 55 | 14 | 553 | 1301 | 12081 | 11560 |
| P2 | CR | W24 | CD8 | 4.40 | 3352787 | 2647520 | 78.96 | 59 | 14 | 542 | 1275 | 11398 | 10939 |
| P3 | CR | BL | CD4 | 9.00 | 4315731 | 3686724 | 85.43 | 58 | 13 | 586 | 1458 | 20536 | 19730 |
| P3 | CR | W12 | CD4 | 3.60 | 4244894 | 3551931 | 83.68 | 57 | 13 | 580 | 1482 | 19995 | 19336 |
| P3 | CR | W24 | CD4 | 7.00 | 4207567 | 3604623 | 85.67 | 57 | 13 | 596 | 1538 | 29509 | 28069 |
| P3 | CR | BL | CD8 | 5.10 | 2163609 | 1777218 | 82.14 | 58 | 13 | 588 | 1392 | 16887 | 16119 |
| P3 | CR | W12 | CD8 | 1.60 | 1914123 | 1564389 | 81.73 | 58 | 13 | 563 | 1308 | 13346 | 12831 |
| P3 | CR | W24 | CD8 | 3.40 | 3015286 | 2391823 | 79.32 | 60 | 13 | 592 | 1436 | 19620 | 18576 |
| P4 | CR | BL | CD4 | 3.10 | 4648893 | 3917473 | 84.27 | 57 | 13 | 576 | 1423 | 16101 | 15390 |
| P4 | CR | W12 | CD4 | 4.60 | 4141022 | 3592976 | 86.77 | 56 | 13 | 565 | 1380 | 12645 | 12129 |
| P4 | CR | W24 | CD4 | 3.90 | 4058012 | 3428462 | 84.49 | 59 | 13 | 592 | 1460 | 16728 | 15982 |
| P4 | CR | BL | CD8 | 3.30 | 2248281 | 1823617 | 81.11 | 55 | 13 | 519 | 1201 | 10175 | 9681 |
| P4 | CR | W12 | CD8 | 3.80 | 1901296 | 1548429 | 81.44 | 59 | 13 | 557 | 1291 | 13098 | 12469 |
| P4 | CR | W24 | CD8 | 3.30 | 2356329 | 1911673 | 81.13 | 59 | 13 | 561 | 1337 | 15924 | 15167 |
| P5 | CR | BL | CD4 | 6.00 | 3076239 | 2567559 | 83.46 | 58 | 13 | 594 | 1533 | 30355 | 28836 |
| P5 | CR | W12 | CD4 | 3.90 | 2556250 | 2169708 | 84.88 | 57 | 13 | 580 | 1422 | 18637 | 17913 |
| P5 | CR | W24 | CD4 | 4.80 | 2733340 | 2323172 | 84.99 | 55 | 13 | 585 | 1447 | 22535 | 21540 |
| P5 | CR | BL | CD8 | 6.80 | 3481705 | 2889960 | 83.00 | 58 | 13 | 601 | 1525 | 28576 | 26923 |
| P5 | CR | W12 | CD8 | 4.80 | 1452880 | 1244217 | 85.64 | 57 | 13 | 545 | 1301 | 13257 | 12730 |
| P5 | CR | W24 | CD8 | 5.00 | 806700 | 685557 | 84.98 | 56 | 13 | 545 | 1301 | 15534 | 14645 |
| P6 | CR | BL | CD4 | 2.90 | 1890183 | 1604343 | 84.88 | 56 | 13 | 567 | 1374 | 16221 | 15596 |
| P6 | CR | W12 | CD4 | 0.95 | 1815738 | 1529744 | 84.25 | 58 | 13 | 510 | 1176 | 7726 | 7562 |
| P6 | CR | W24 | CD4 | 8.50 | 2123051 | 1775571 | 83.63 | 59 | 13 | 576 | 1408 | 18690 | 17858 |
| P6 | CR | BL | CD8 | 1.20 | 3120418 | 2291615 | 73.44 | 58 | 13 | 524 | 1211 | 9194 | 8926 |
| P6 | CR | W12 | CD8 | 0.38 | 3376465 | 2629360 | 77.87 | 55 | 13 | 529 | 1229 | 9188 | 8954 |
| P6 | CR | W24 | CD8 | 3.00 | 2815008 | 2094130 | 74.39 | 58 | 13 | 543 | 1291 | 13170 | 12572 |
| P7 | CR | BL | CD4 | 5.70 | 5595286 | 4816597 | 86.08 | 57 | 13 | 592 | 1481 | 21581 | 20609 |
| P7 | CR | W12 | CD4 | 5.50 | 3716158 | 3197076 | 86.03 | 58 | 13 | 580 | 1425 | 17007 | 16598 |
| P7 | CR | W24 | CD4 | 6.30 | 4523201 | 3844411 | 84.99 | 57 | 13 | 588 | 1483 | 23613 | 22793 |
| P7 | CR | BL | CD8 | 9.70 | 3595047 | 2815047 | 78.30 | 61 | 13 | 604 | 1533 | 31309 | 29442 |
| P7 | CR | W12 | CD8 | 7.30 | 1620004 | 1298612 | 80.16 | 56 | 13 | 543 | 1268 | 12741 | 12177 |
| P7 | CR | W24 | CD8 | 9.90 | 3426367 | 2747287 | 80.18 | 59 | 13 | 605 | 1542 | 26712 | 25444 |
| P8 | CR | BL | CD4 | 5.30 | 2913917 | 2379288 | 81.65 | 57 | 13 | 593 | 1564 | 23293 | 22267 |
| P8 | CR | W12 | CD4 | 2.40 | 2873584 | 2369129 | 82.45 | 57 | 14 | 540 | 1274 | 10107 | 9940 |
| P8 | CR | W24 | CD4 | 2.80 | 2481366 | 2027689 | 81.72 | 59 | 14 | 583 | 1510 | 20655 | 19800 |
| P8 | CR | BL | CD8 | 5.10 | 2550515 | 1943939 | 76.22 | 61 | 14 | 599 | 1502 | 23862 | 22395 |
| P8 | CR | W12 | CD8 | 2.70 | 2620235 | 2119693 | 80.90 | 59 | 14 | 553 | 1334 | 13329 | 12848 |
| P8 | CR | W24 | CD8 | 2.10 | 2948088 | 2377041 | 80.63 | 56 | 13 | 552 | 1316 | 14525 | 13967 |
| P9 | CR | BL | CD4 | 6.36 | 3222444 | 2639521 | 81.91 | 62 | 13 | 679 | 1831 | 81531 | 76221 |
| P9 | CR | W12 | CD4 | 14.60 | 3393537 | 2871313 | 84.61 | 60 | 13 | 661 | 1746 | 66027 | 62314 |
| P9 | CR | W24 | CD4 | 14.30 | 2492427 | 2133980 | 85.62 | 60 | 13 | 590 | 1478 | 23798 | 23316 |
| P9 | CR | BL | CD8 | 7.27 | 1315785 | 723189 | 54.96 | 61 | 13 | 646 | 1678 | 69585 | 63082 |
| P9 | CR | W12 | CD8 | 14.40 | 799747 | 437446 | 54.70 | 57 | 13 | 640 | 1665 | 76408 | 70756 |
| P9 | CR | W24 | CD8 | 13.00 | 1197930 | 527031 | 44.00 | 60 | 13 | 633 | 1664 | 82408 | 76725 |
| P10 | CR | BL | CD4 | 5.56 | 2029326 | 1752543 | 86.36 | 58 | 13 | 576 | 1430 | 16995 | 16545 |
| P10 | CR | W12 | CD4 | 14.60 | 2412695 | 2099545 | 87.02 | 60 | 13 | 631 | 1637 | 32999 | 31579 |
| P10 | CR | W24 | CD4 | 5.67 | 3307101 | 2849766 | 86.17 | 57 | 13 | 625 | 1639 | 36160 | 34681 |
| P10 | CR | BL | CD8 | 5.78 | 1352845 | 740052 | 54.70 | 59 | 13 | 626 | 1633 | 78576 | 71045 |
| P10 | CR | W12 | CD8 | 13.30 | 1247233 | 717784 | 57.55 | 62 | 13 | 661 | 1777 | 169385 | 153930 |
| P10 | CR | W24 | CD8 | 6.00 | 590157 | 339464 | 57.52 | 54 | 13 | 442 | 920 | 4438 | 4170 |
| NP1 | NCR | BL | CD4 | 3.70 | 2154483 | 1814798 | 84.23 | 58 | 13 | 598 | 1540 | 29727 | 27980 |
| NP1 | NCR | W12 | CD4 | 3.20 | 3234989 | 2725762 | 84.26 | 56 | 13 | 556 | 1321 | 13707 | 13202 |
| NP1 | NCR | W24 | CD4 | 5.10 | 2946120 | 2471717 | 83.90 | 56 | 13 | 567 | 1386 | 18446 | 17691 |
| NP1 | NCR | BL | CD8 | 1.30 | 4249549 | 3471128 | 81.68 | 57 | 13 | 553 | 1331 | 13848 | 13238 |
| NP1 | NCR | W12 | CD8 | 1.10 | 3164719 | 2607959 | 82.41 | 58 | 13 | 539 | 1246 | 10025 | 9702 |
| NP1 | NCR | W24 | CD8 | 3.00 | 2133042 | 1721339 | 80.70 | 58 | 13 | 512 | 1166 | 8978 | 8693 |
| NP2 | NCR | BL | CD4 | 3.70 | 3201563 | 2597945 | 81.15 | 59 | 13 | 622 | 1605 | 33769 | 31878 |
| NP2 | NCR | W12 | CD4 | 2.10 | 3557127 | 2937278 | 82.57 | 59 | 13 | 590 | 1479 | 19164 | 18471 |
| NP2 | NCR | W24 | CD4 | 3.40 | 1848355 | 1519144 | 82.19 | 58 | 13 | 585 | 1400 | 16153 | 15500 |
| NP2 | NCR | BL | CD8 | 3.40 | 2425778 | 1935386 | 79.78 | 61 | 13 | 588 | 1425 | 17612 | 16725 |
| NP2 | NCR | W12 | CD8 | 2.00 | 2909579 | 2351512 | 80.82 | 60 | 13 | 546 | 1286 | 13160 | 12549 |
| NP2 | NCR | W24 | CD8 | 3.40 | 3713269 | 2928204 | 78.86 | 58 | 13 | 563 | 1337 | 13970 | 13397 |
| NP3 | NCR | BL | CD4 | 0.47 | 2287925 | 1770052 | 77.36 | 53 | 13 | 521 | 1197 | 7758 | 7568 |
| NP3 | NCR | W12 | CD4 | 2.28 | 2420192 | 2054639 | 84.90 | 58 | 13 | 590 | 1485 | 20747 | 19810 |
| NP3 | NCR | W24 | CD4 | 4.10 | 2869504 | 2388893 | 83.25 | 58 | 13 | 579 | 1441 | 18498 | 17689 |
| NP3 | NCR | BL | CD8 | 0.27 | 2322168 | 1802925 | 77.64 | 52 | 13 | 474 | 1034 | 5081 | 4995 |
| NP3 | NCR | W12 | CD8 | 2.55 | 3437516 | 2747538 | 79.93 | 57 | 13 | 511 | 1191 | 8745 | 8508 |
| NP3 | NCR | W24 | CD8 | 2.90 | 2899268 | 2280926 | 78.67 | 56 | 13 | 510 | 1193 | 8135 | 7888 |
| NP4 | NCR | BL | CD4 | 1.80 | 4051382 | 3359181 | 82.91 | 58 | 13 | 598 | 1519 | 22835 | 22058 |
| NP4 | NCR | W12 | CD4 | 5.10 | 5332551 | 4514698 | 84.66 | 56 | 13 | 596 | 1485 | 22709 | 21905 |
| NP4 | NCR | W24 | CD4 | 4.00 | 4832800 | 4093654 | 84.71 | 57 | 13 | 601 | 1505 | 24046 | 23100 |
| NP4 | NCR | BL | CD8 | 2.30 | 1890280 | 1551822 | 82.09 | 55 | 13 | 568 | 1392 | 16660 | 15962 |
| NP4 | NCR | W12 | CD8 | 4.30 | 2212503 | 1782818 | 80.58 | 56 | 13 | 507 | 1107 | 6430 | 6179 |
| NP4 | NCR | W24 | CD8 | 5.80 | 2396485 | 1835111 | 76.58 | 56 | 13 | 560 | 1344 | 14088 | 13426 |
| NP5 | NCR | BL | CD4 | 5.70 | 2931140 | 2450595 | 83.61 | 60 | 13 | 626 | 1605 | 33812 | 31852 |
| NP5 | NCR | W12 | CD4 | 3.60 | 2764538 | 2343678 | 84.78 | 61 | 13 | 630 | 1600 | 32405 | 30555 |
| NP5 | NCR | W24 | CD4 | 3.60 | 3149484 | 2656029 | 84.33 | 58 | 13 | 603 | 1516 | 23582 | 22453 |
| NP5 | NCR | BL | CD8 | 7.50 | 2227271 | 1834337 | 82.36 | 58 | 13 | 556 | 1361 | 16874 | 16113 |
| NP5 | NCR | W12 | CD8 | 6.10 | 2453407 | 2036840 | 83.02 | 60 | 13 | 596 | 1460 | 20743 | 19786 |
| NP5 | NCR | W24 | CD8 | 4.50 | 2825192 | 2322869 | 82.22 | 57 | 13 | 569 | 1353 | 16369 | 15666 |
| NP6 | NCR | BL | CD4 | 5.50 | 4636485 | 3975444 | 85.74 | 59 | 13 | 614 | 1564 | 31499 | 29965 |
| NP6 | NCR | W12 | CD4 | 7.10 | 5425043 | 4577022 | 84.37 | 59 | 13 | 632 | 1613 | 32907 | 31190 |
| NP6 | NCR | W24 | CD4 | 3.40 | 4393668 | 3662474 | 83.36 | 55 | 13 | 578 | 1467 | 21451 | 20798 |
| NP6 | NCR | BL | CD8 | 2.40 | 3026588 | 2369725 | 78.30 | 59 | 13 | 607 | 1506 | 23686 | 22347 |
| NP6 | NCR | W12 | CD8 | 1.50 | 1928281 | 1571777 | 81.51 | 59 | 13 | 561 | 1315 | 11961 | 11545 |
| NP6 | NCR | W24 | CD8 | 1.10 | 2924473 | 2370568 | 81.06 | 58 | 13 | 552 | 1316 | 11842 | 11535 |
| NP7 | NCR | BL | CD4 | 4.80 | 3046511 | 2587590 | 84.94 | 60 | 13 | 590 | 1480 | 23338 | 22196 |
| NP7 | NCR | W12 | CD4 | 7.00 | 8258220 | 6853546 | 82.99 | 59 | 13 | 613 | 1584 | 30269 | 28731 |
| NP7 | NCR | W24 | CD4 | 5.60 | 6661407 | 5643114 | 84.71 | 59 | 13 | 614 | 1586 | 33497 | 31682 |
| NP7 | NCR | BL | CD8 | 3.20 | 3150143 | 2627343 | 83.40 | 58 | 13 | 571 | 1359 | 12041 | 11625 |
| NP7 | NCR | W12 | CD8 | 4.00 | 3074621 | 2385611 | 77.59 | 58 | 13 | 557 | 1334 | 11064 | 10717 |
| NP7 | NCR | W24 | CD8 | 3.60 | 3058721 | 2488189 | 81.35 | 59 | 13 | 570 | 1366 | 14324 | 13677 |
| NP8 | NCR | BL | CD4 | 4.80 | 4561542 | 3887481 | 85.22 | 59 | 13 | 593 | 1483 | 20379 | 19413 |
| NP8 | NCR | W12 | CD4 | 10.00 | 1982620 | 1678757 | 84.67 | 61 | 13 | 617 | 1568 | 28605 | 26950 |
| NP8 | NCR | W24 | CD4 | 1.40 | 2620502 | 2172535 | 82.91 | 59 | 13 | 560 | 1292 | 9903 | 9458 |
| NP8 | NCR | BL | CD8 | 2.20 | 1440483 | 1159975 | 80.53 | 59 | 13 | 532 | 1191 | 7942 | 7597 |
| NP8 | NCR | W12 | CD8 | 4.60 | 1791640 | 1443263 | 80.56 | 60 | 13 | 583 | 1434 | 17468 | 16553 |
| NP8 | NCR | W24 | CD8 | 0.71 | 2036491 | 1630618 | 80.07 | 56 | 13 | 501 | 1064 | 5832 | 5628 |
| NP9 | NCR | BL | CD4 | 3.50 | 2549001 | 2193868 | 86.07 | 56 | 14 | 523 | 1223 | 8046 | 7882 |
| NP9 | NCR | W12 | CD4 | 3.50 | 2553018 | 2183379 | 85.52 | 59 | 13 | 598 | 1522 | 24853 | 23633 |
| NP9 | NCR | W24 | CD4 | 2.80 | 2672103 | 2276923 | 85.21 | 58 | 13 | 576 | 1391 | 14648 | 14109 |
| NP9 | NCR | BL | CD8 | 4.80 | 1584807 | 1149820 | 72.55 | 56 | 13 | 517 | 1177 | 8849 | 8404 |
| NP9 | NCR | W12 | CD8 | 5.00 | 1440577 | 1121876 | 77.88 | 58 | 13 | 547 | 1242 | 9426 | 8967 |
| NP9 | NCR | W24 | CD8 | 3.40 | 1507496 | 1118083 | 74.17 | 57 | 13 | 465 | 969 | 4363 | 4203 |
| NP10 | NCR | BL | CD4 | 3.60 | 7458597 | 6420460 | 86.08 | 60 | 13 | 617 | 1586 | 29566 | 28098 |
| NP10 | NCR | W12 | CD4 | 3.40 | 6880495 | 5863927 | 85.23 | 59 | 13 | 610 | 1572 | 31148 | 29510 |
| NP10 | NCR | W24 | CD4 | 3.70 | 6338563 | 5446161 | 85.92 | 57 | 13 | 600 | 1534 | 25216 | 24128 |
| NP10 | NCR | BL | CD8 | 5.20 | 3382987 | 2852372 | 84.32 | 58 | 13 | 596 | 1451 | 17873 | 17249 |
| NP10 | NCR | W12 | CD8 | 3.50 | 3038982 | 2566924 | 84.47 | 59 | 13 | 593 | 1472 | 18554 | 17864 |
| NP10 | NCR | W24 | CD8 | 2.70 | 2287657 | 1855334 | 81.10 | 59 | 13 | 567 | 1328 | 11483 | 10977 |
| NP11 | NCR | BL | CD4 | 6.14 | 2788355 | 2378433 | 85.30 | 58 | 13 | 630 | 1672 | 48523 | 46254 |
| NP11 | NCR | W12 | CD4 | 14.00 | 3024484 | 2578719 | 85.26 | 61 | 13 | 670 | 1788 | 86738 | 80370 |
| NP11 | NCR | W24 | CD4 | 8.00 | 2622720 | 2219233 | 84.62 | 60 | 13 | 623 | 1635 | 47910 | 45769 |
| NP11 | NCR | BL | CD8 | 6.86 | 1131518 | 661430 | 58.46 | 60 | 13 | 607 | 1565 | 43139 | 38809 |
| NP11 | NCR | W12 | CD8 | 10.10 | 1312240 | 706022 | 53.80 | 59 | 13 | 638 | 1686 | 100396 | 91200 |
| NP11 | NCR | W24 | CD8 | 7.35 | 989095 | 572169 | 57.85 | 59 | 13 | 637 | 1677 | 88153 | 79416 |
| NP12 | NCR | BL | CD4 | 15.30 | 3488727 | 3004407 | 86.12 | 59 | 13 | 626 | 1636 | 40919 | 38917 |
| NP12 | NCR | W12 | CD4 | 8.27 | 2463967 | 2125556 | 86.27 | 60 | 13 | 664 | 1779 | 72161 | 66472 |
| NP12 | NCR | W24 | CD4 | 14.60 | 1749909 | 1511466 | 86.37 | 59 | 13 | 650 | 1740 | 76504 | 68844 |
| NP12 | NCR | BL | CD8 | 9.70 | 1095499 | 575389 | 52.52 | 59 | 13 | 563 | 1339 | 14351 | 13198 |
| NP12 | NCR | W12 | CD8 | 10.80 | 931857 | 487370 | 52.30 | 59 | 13 | 643 | 1701 | 80990 | 76514 |
| NP12 | NCR | W24 | CD8 | 10.80 | 1009132 | 519325 | 51.46 | 57 | 13 | 642 | 1702 | 81646 | 76292 |

Clean reads, number of filter-passing raw data reads; Total productive TCRβ sequence reads, total number of TCRβ sequence reads which are expected to translate to functional TCR proteins; Productive TCRβ reads (%) is calculated as the number of total productive TCRβ reads/clean reads; Unique CDR3 nt clonotype, number of unique nucleotide sequence in the CDR3 region; Unique CDR3 aa clonotype, the number of identical CDR3 amino acid sequences.
