# Supplementary figures and images for: Dynamic Perturbations of CD4 and CD8 T Cell Receptor Repertoires in Chronic Hepatitis B Patients upon Oral Antiviral Therapy
Source: Front Immunol. 2017 Sep 14;8:1142. doi: 10.3389/fimmu.2017.01142 (PMC5603711; doi:10.3389/fimmu.2017.01142)

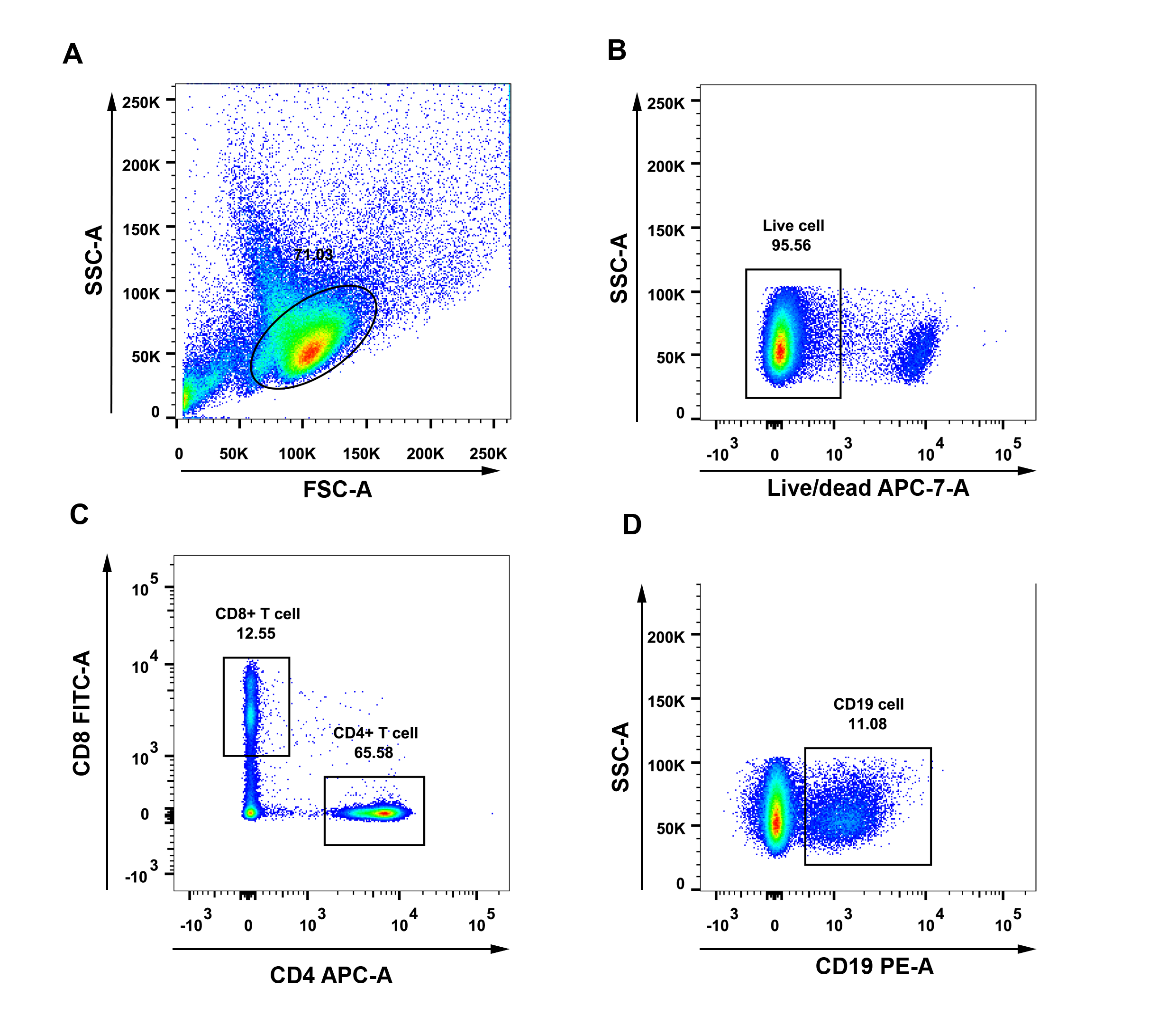

Supplement: Figure S1 — Flow cytometric cell-sorting strategy for isolation of CD4+, CD8+ T cells and CD19+ cells. CD4+, CD8+ T cells and CD19+ cells were identified by gating on lymphocytes (A), followed by exclusion of dead cells (B) and selection of CD4+, CD8+ T cells (C) and CD19+ cells (D). The figures show representative FACS profiles of one chronic hepatitis B (CHB) patients and the strategy used for sorting. [file Image_1.TIF]

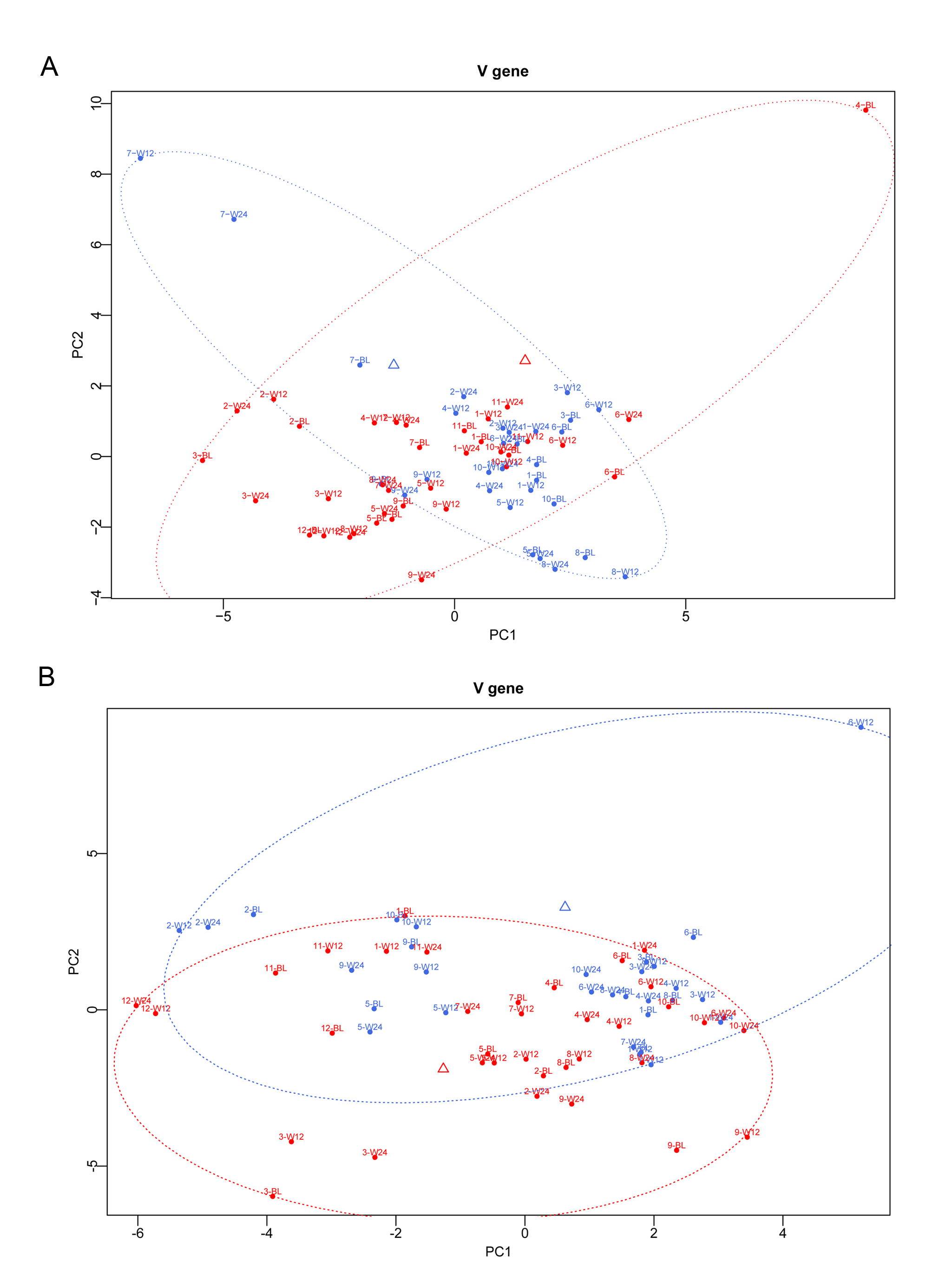

Supplement: Figure S4 — Principal component analysis of Vβ gene segment usage in CD4 and CD8 T cells. Principal component analysis shows the usage patterns of Vβ gene segments of CD4 (A) and CD8 (B) samples of complete response (CR) group patients (red dots) and non-complete response (NCR) group patients (blue dots). PC1 refers to the first principal component, whereas PC2 indicates the second principal component. [file Image_4.TIFF]

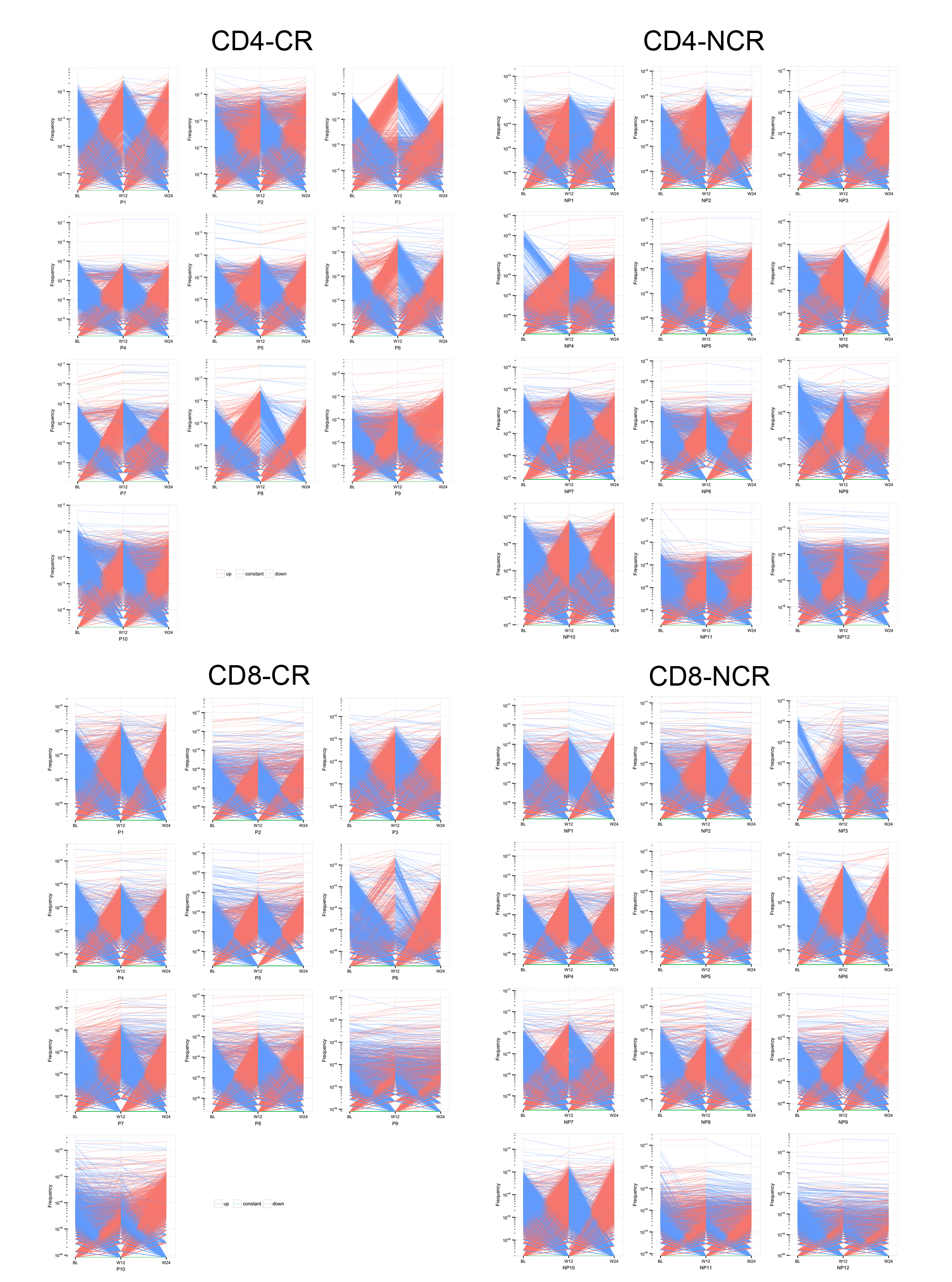

Supplement: Figure S5 — Fluctuations of overall CD4 and CD8 clone repertoires in different groups. Longitudinal data points from each clonotype in each patient are connected by a line (frequency increased, red line; frequency decreased, blue line, frequency constant, green line). The log10-transformed frequency is indicated on the y-axis. The x-axis represents treatment timepoints. BL, baseline; W12, week 12; W24, week 24. [file Image_5.TIF]

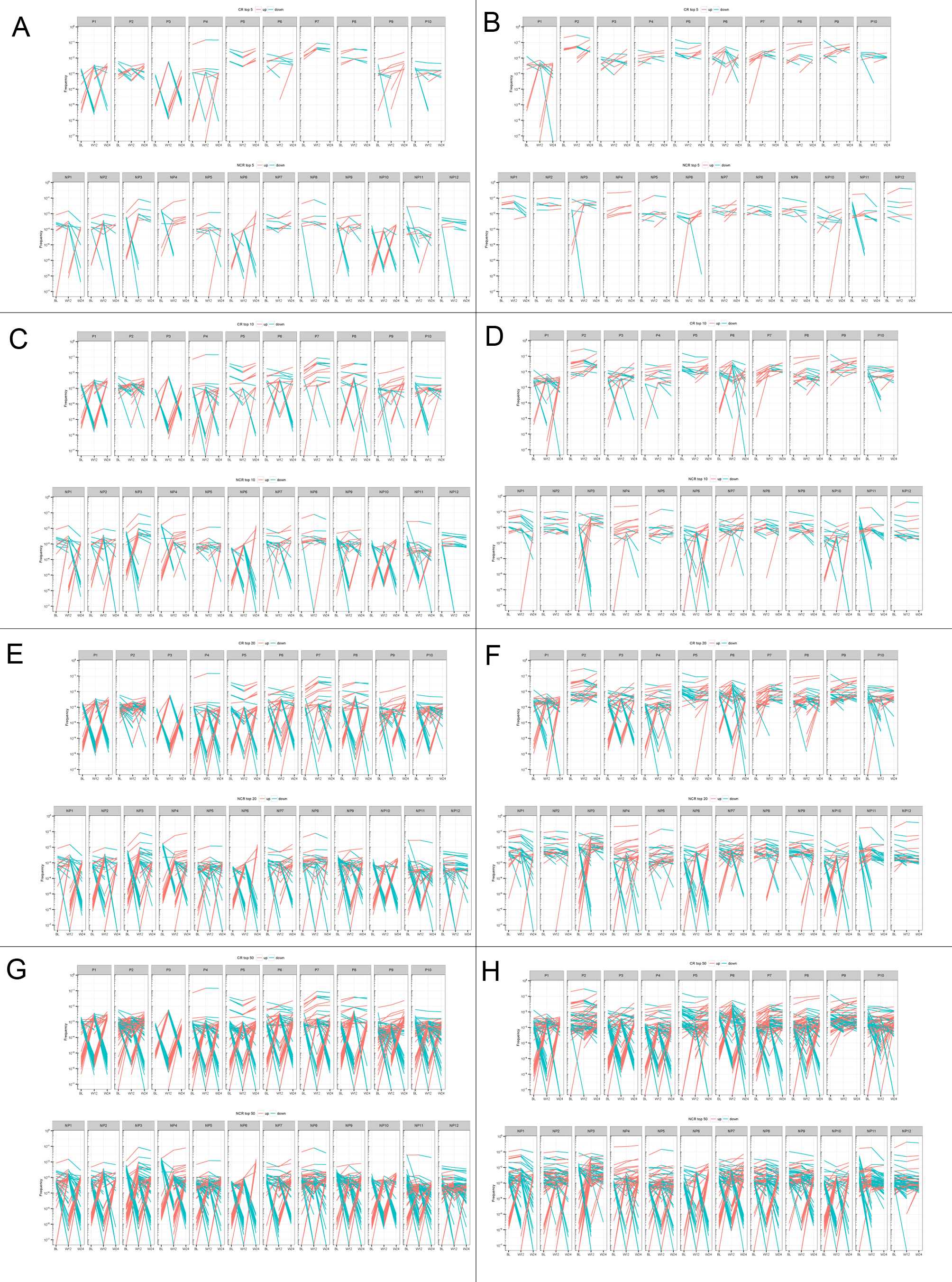

Supplement: Figure S6 — Tracking the dominant clones over the treatment. The top5 (A,B), top 10 (C,D), top 20 (E,F), top 50 (G,H) clonotypes in CD4 (left) and CD8 (right) subsets in each patient [complete response (CR) group, upper lane; non-complete response (NCR) group, lower lane] are listed, respectively, and tracking them according to their frequency (y-axis) at baseline, week 12, and 24 (x-axis). Identical clonotypes are connected by a line (frequency increased, red line; frequency decreased, green line). [file Image_6.TIF]
